# Earthy Deposit in the Cornea

**Published:** 1881-03

**Authors:** S. J. Parker

**Affiliations:** Ithaca, N. Y.


ARTICLE III.

### EARTHY DEPOSIT IN THE CORNEA.

BY S. J. PARKER, M. D., ITHACA, N. Y.

Tyrell's " Diseases of the Eye " is an old book, which justly is esteemed, because it has less of extravagant nomenclature for the anatomy of the eye, and more common sense description than later voluminous works. He says, Vol. 1, Page. 246, Ed. 1840, that he has seen several cases of earthy deposit in the cornea; which in one case was an eighth of an inch in diameter. A year or two ago, Mr.——, an old man, remarkable for his pugnacious spirit called on me to see if I could "restore his sight." Both eyes were nebulous in their cornea. The left one hopelessly so, the right with a flabby bag enclosing a solid substance, nearly the size of the cornea. He said it is a stone, I can feel it grit; putting his finger to the globe, he held it like a small button, and produced a gritty sensation as he rubbed the large mass against gritty grains in the bag. And said: "Doctor, if that was cut off, I could see; I see best with that eye." Meaning that there was misty light seen in the looseness about the stony (earthy) matter. I said I can cut the bag open, remove the stony matter; and the eye will feel easier. He replied, "Cut away doctor, but I shall hold you responsible if I cannot see." I then said, you can never see again with either eye. All I can do is to make them less sore or painful. And since you threaten to sue, if I cannot restore sight, I cannot do anything for you, not even relieve your pain. And with this I dismissed him, and lost sight of him for months, when one day he came bringing in a paper a piece of limelike substance, saying: "I kept working at it until I got it out, here it is." There was an excavation in the eye, as if cornea, iris and lens were gone, the inflammation was gone, and he wished an operation to enable him to see. I told him no operation could do that, when his wife whispered, "Don't touch his eye. All he wants is a law-suit." So I merely looked at the eye, and sent him away, with the injunction to call no more. This was a case of earthy (lime) deposit, such as would occupy the whole of the aqueous chamber, pushing back the iris, and followed by the loss of the cornea. How it began I cannot say, but probably in a ceratitis, which was neglected, as no one wishes to treat him since a suit, years ago, for fractured limb, and his well-known unreasonable character. It is the largest accumulation of whitish yellow earthy matter I have ever seen, or read of.

